# Endothelial Function Is Preserved in Patients with Wild-Type Transthyretin Amyloid Cardiomyopathy

**DOI:** 10.3390/jcm12072534

**Published:** 2023-03-27

**Authors:** Yu Hashimoto, Takayuki Yamaji, Toshiro Kitagawa, Yukiko Nakano, Masato Kajikawa, Kenichi Yoshimura, Kazuaki Chayama, Chikara Goto, Syunsuke Tanigawa, Aya Mizobuchi, Takahiro Harada, Farina Mohamad Yusoff, Shinji Kishimoto, Tatsuya Maruhashi, Asuka Fujita, Toshio Uchiki, Ayumu Nakashima, Yukihito Higashi

**Affiliations:** 1 Department of Cardiovascular Medicine, Graduate School of Biomedical Sciences, Hiroshima University, Hiroshima 734-8551, Japan; 2 Division of Regeneration and Medicine, Medical Center for Translational and Clinical Research, Hiroshima University Hospital, Hiroshima 734-8551, Japan; 3 Department of Biostatistics, Medical Center for Translational and Clinical Research, Hiroshima University Hospital, Hiroshima 734-8551, Japan; 4 Collaborative Research Laboratory of Medical Innovation, Hiroshima University, Hiroshima 734-8551, Japan; 5 Department of Physical Therapy, Hiroshima International University, Hiroshima 737-0112, Japan; 6 Department of Regenerative Medicine, Research Institute for Radiation Biology and Medicine, Hiroshima University, Hiroshima 739-8529, Japan; 7 Plastic and Reconstructive Surgery, Hiroshima University Hospital, Hiroshima 734-8551, Japan; 8 Department of Stem Cell Biology and Medicine, Graduate School of Biomedical and Health Sciences, Hiroshima University, Hiroshima 734-8551, Japan

**Keywords:** endothelial function, flow-mediated vasodilation, wild-type transthyretin amyloidosis cardiomyopathy, N-terminal pro-brain natriuretic peptide

## Abstract

Heart failure (HF) is associated with endothelial dysfunction. Vascular function per se plays an important role in cardiac function, whether it is a cause or consequence. However, there is no information on vascular function in patients with wild-type transthyretin amyloid cardiomyopathy (ATTRwt-CM). The purpose of this study was to evaluate vascular function in patients with ATTRwt-CM. We measured flow-mediated vasodilation (FMD) as an index of endothelial function and nitroglycerine-induced vasodilation (NID) as an index of vascular smooth muscle function and brachial artery intima-media thickness (bIMT) and brachial-ankle pulse wave velocity (baPWV) as indices of arterial stiffness in 22 patients with ATTRwt-CM and in 22 one-by-one matched control patients using vascular function confounding factors. FMD was significantly greater in patients with ATTRwt-CM than in the controls (5.4 ± 3.4% versus 3.5 ± 2.4%, *p* = 0.038) and the N-terminal pro-brain natriuretic peptide (NT-proBNP) level was significantly greater in patients with ATTRwt-CM than in the controls (2202 ± 1478 versus 470 ± 677 pg/mL, *p* < 0.001). There were no significant differences in NID, bIMT or baPWV between the two groups. There was a significant relationship between NT-proBNP and FMD in patients with ATTRwt-CM (r = 0.485, *p* = 0.022). NT-proBNP showed no significant relationships with NID, bIMT or baPWV. Conclusions: Endothelial function was preserved in patients with ATTRwt-CM. Patients with ATTRwt-CM may have compensatory effects with respect to endothelial function through elevation of BNP.

## 1. Introduction

Transthyretin amyloid cardiomyopathy (ATTR-CM) is caused by extracellular deposits of abnormal fibrils derived from transthyretin (TTR) protein, which is mainly produced by the liver [1,2]. The abnormal fibrils aggregate and are deposited in various organs including the heart, carpus, spine, arteries, and nerves [1,2]. ATTR-CM is classified into two subtypes according to genetic mutation in the TTR gene: hereditary transthyretin amyloidosis (hATTR), alias ATTR variant, and wild-type transthyretin amyloidosis with no mutation (ATTRwt) [1,2,3,4]. Although ATTR-CM has been considered a rare disease, previous autopsy studies revealed that ATTRwt-CM was present in 12~15% of subjects over 80 years of age [5,6], suggesting that ATTRwt-CM is under-recognized [1,7]. Recently, with advances in imaging capabilities and increased recognition by cardiologists, more patients with ATTRwt-CM are being diagnosed [3,8]. It has been revealed that the prognosis of ATTRwt-CM is poor with a median overall survival period of 58.9 months after diagnosis and that 79% of the deaths were due to cardiovascular disease [9].

It has been known since as early as the 1990s that heart failure (HF) is associated with endothelial dysfunction [10]. We have also clearly shown that endothelial function is impaired in patients with dilated cardiomyopathy and in patients with heart failure who have a preserved ejection fraction [11,12]. Endothelial dysfunction is an independent risk factor of cardiovascular events [13,14]. In patients with HF, endothelial function is also a predictor of cardiovascular events [15]. In addition, vascular function including endothelial function and vascular smooth muscle function per se plays an important role in cardiac function, whether it is a cause or consequence.

In addition to poor prognosis, cardiac amyloidosis is characterized by a restrictive cardiomyopathy [16]. Although beta blockers, angiotensin-converting enzyme inhibitors and angiotensin receptor blockers are commonly used in patients with congestive HF, those drugs may have harmful effects in patients with ATTR-CM [17]. Because of these many differences, we need to distinguish ATTR-CM from common HF. There is no information on vascular function in patients with ATTR-CM. Therefore, we measured flow-mediated vasodilation (FMD) as an index of endothelial function and nitroglycerine-induced vasodilation (NID) as an index of vascular smooth muscle function by using a high-resolution ultrasound system and we measured brachial artery intima-media thickness (bIMT) and brachial-ankle pulse wave velocity (baPWV) as indices of arterial stiffness in patients with ATTRwt-CM.

## 2. Materials and Methods

### 2.1. Subjects

Between July 2007 and May 2021, a total of 3966 patients were recruited for the measurement of vascular function from subjects who underwent a health-screening examination or who visited the outpatient clinic at Hiroshima University Hospital. We excluded the following patients: patients being treated with nitrate, patients with types of amyloidosis other than ATTRwt-CM, and patients with ATTRwt-CM who had severe congestive HF (New York Heart Association (NYHA) level of Ⅳ). Of those patients, 22 patients had ATTRwt-CM (2 patients with NYHA I, 5 patients with NYHA II, and 15 patients with NYHA Ⅲ). Three patients with NYHA Ⅳ were excluded from this study. In all of the patients with ATTRwt-CM, an endomyocardial biopsy was performed to demonstrate disease-specific deposition and a blood test was performed to confirm that there was no TTR gene mutation. All of the patients with ATTRwt-CM underwent blood tests and vascular function tests with adequate supportive care, and the patients were not in the decompensated phase of HF. Hypertension was defined by treatment with oral antihypertensive agents or by systolic blood pressure of more than 140 mmHg or diastolic blood pressure of more than 90 mmHg when measured in a sitting position on at least 3 different occasions. *Diabetes mellitus* was defined according to the American Diabetes Association recommendations [18]. Dyslipidemia was defined according to the third report of the National Cholesterol Education Program [19]. Smokers were defined as subjects who had smoked for at least one year and had smoked more than 100 cigarettes in their lifetime. Coronary heart disease included angina pectoris, myocardial infarction, and unstable angina. Cerebrovascular disease included ischemic stroke, hemorrhagic stroke, and transient ischemic attack. Cardiovascular disease was defined as coronary heart disease and cerebrovascular disease. The Ethics Review Board of Hiroshima University approved the study protocol (UMIN0000039512). Written informed consent for participation in the study was obtained from all of the subjects.

### 2.2. Study Protocol

We measured vascular responses to reactive hyperemia and we also measured bIMT in all of the subjects. Echocardiograms were also obtained from all of the subjects. The subjects fasted the previous night for at least 12 h prior to the measurements. The study began at 8:30 a.m. The subjects were kept in the supine position in a quiet, dark, air-conditioned room (constant temperature of 22–25 °C) throughout the study. A 23-gauge polyethylene catheter was inserted into the left deep antecubital vein to obtain blood samples. After thirty minutes of the subjects maintaining the supine position, their FMD, bIMT, NID, and baPWV were measured. The observers were blind to the form of examination. Thirty minutes after remaining in the supine position, fasting serum concentrations of total cholesterol, high-density lipoprotein cholesterol (HDL-C), low-density lipoprotein cholesterol (LDL-C), triglycerides, glucose, and N-terminal pro-brain natriuretic peptide (NT-proBNP) were measured.

### 2.3. Measurements of FMD and NID

Vascular response to hyperemia in the brachial artery was used for the assessment of endothelium-dependent FMD. A high-resolution liner artery transducer was coupled with computer-assisted analysis software (UNEXEF18G, UNEX Co., Nagoya, Japan) that used an automated edge detection system for the measurement of brachial artery diameter [20]. The response to nitroglycerine was used for assessment of endothelium-independent vasodilation. NID was measured as described previously [20]. Please see the online data supplement for additional details.

### 2.4. Measurement of Brachial IMT

Before FMD measurement, baseline longitudinal ultrasonographic images of the brachial artery, obtained at the end of diastole from each of 10 cardiac cycles, were automatically stored on a hard disk for off-line assessment of IMT with a linear, phased-array high-frequency (10-MHz) transducer using an UNEXEF18G ultrasound unit (UNEX Co.). Please see the online data supplement for additional details.

### 2.5. Measurement of baPWV

Aortic compliance was assessed noninvasively on the basis of Doppler ultrasound measurements of PWV along the descending thoracoabdominal aorta, as previously published and validated. Please see the online data supplement for additional details.

### 2.6. Echocardiography

Echocardiograms were obtained by using commercially available equipment (Vivid E95, GE). Please see the online data supplement for additional details.

### 2.7. Statistical Analysis

Results are presented as means ± SD for continuous variables and as percentages for categorical variables. All reported probability values were 2-sided, and a probability value of <0.05 was considered statistically significant. Continuous variables were compared by using ANOVA. Categorical variables were compared by means of the χ^2^ test. One-to-one propensity-score matching analyses were used to create matched pairs in order to investigate the association between ATTRwt-CM and endothelial function. The propensity score was calculated for each patient on the basis of logistic regression analysis of the probability of ATTRwt-CM including age, sex, body mass index, heart rate, systolic blood pressure, diastolic blood pressure, total cholesterol, HDL-C, LDL-C, glucose, hemoglobin A1C, estimated glomerular filtration rate, hypertension (yes/no), dyslipidemia (yes/no), diabetes mellitus (yes/no), hyperuricemia (yes/no), and current smoker (yes/no). With these propensity scores, two well-matched groups based on clinical characteristics were created with a caliper width of 0.02 for comparison of vascular function. Relationships between variables were determined by Pearson correlation coefficients analysis. In addition, we created matched pairs (1 patient with ATTRwt-CM to 1 control) using vascular function confounding factors including age, sex, body mass index, heart rate, systolic blood pressure, diastolic blood pressure, total cholesterol, triglycerides, HDL-C, LDL-C, glucose, hemoglobin A1c, estimated glomerular filtration rate, hypertension, dyslipidemia, diabetes mellitus, hyperuricemia, and current smoking. The paired *t*-test was used for comparison of mean values of continuous variables between the two groups. Correlations between continuous variables were estimated with the use of Pearson correlation coefficients. All analyses were conducted using JMP version 15.0 software (SAS Institute, Cary, NC, USA).

## 3. Results

### 3.1. Baseline Characteristics

The baseline clinical characteristics of the 3792 patients without ATTRwt-CM and 22 patients with ATTRwt-CM are summarized in Supplementary Table S1. Age, HDL-C, and uric acid were significantly higher in patients with ATTRwt-CM than in patients without ATTRwt-CM. Systolic blood pressure, LDL-C, prevalence of hypertension, and frequency of use of calcium channel blockers were significantly lower in patients with ATTRwt-CM than in patients without ATTRwt-CM. There were no significant differences in other parameters between patients without ATTRwt-CM and patients with ATTRwt-CM.

Next, because of the different clinical characteristics, we evaluated vascular function in patients with ATTRwt-CM and control patients using the propensity score matching method to make matched pairs between patients with ATTRwt-CM and controls based on vascular function confounding factors from the 3792 patients without ATTRwt-CM.

The baseline clinical characteristics of the 22 matched control patients without ATTRwt-CM and 22 patients with ATTRwt-CM are summarized in Table 1. Serum concentration of NT-proBNP was significantly higher in patients with ATTRwt-CM than in the controls. There was no significant difference in other parameters between the two groups. Echocardiographic parameters are summarized in Supplementary Table S2. Left Ventricular ejection fraction (LVEF) was not significantly different between the two groups, but the number of patients with LVEF <50% was significantly greater in the ATTRwt-CM group than in the control group. Left atrium volume index, interventricular septal thickness, posterior LV wall thickness, E/A, and E/e’ were significantly greater in the ATTRwt-CM group than in the control group.

### 3.2. Vascular Function

**Figure 1:**
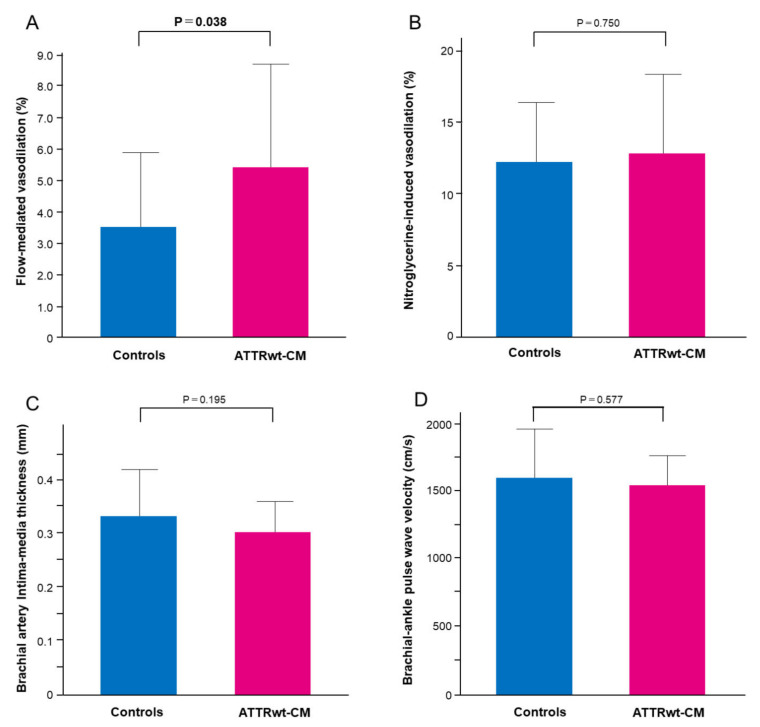
Comparison of flow-mediated vasodilation (FMD), nitroglycerine-induced vasodilation (NID), brachial intima-media thickness (bIMT) and brachial-ankle pulse wave velocity (baPWV) between controls and wild-type transthyretin amyloid cardiomyopathy (ATTRwt-CM). Bar graphs show FMD (**A**) and NID (**B**), bIMT (**C**) and baPWV (**D**) in controls and patients with ATTRwt-CM.

FMD was significantly greater in patients with ATTRwt-CM than in one-by-one matched controls (5.4 ± 3.4% versus 3.5 ± 2.4%, *p* = 0.038) (Figure 1A). Vascular responses to nitroglycerine were not significantly different between the two groups (12.8 ± 5.6% versus 12.3 ± 4.3%, *p* = 0.750) (Figure 1B). The values of bIMT and baPWV were not significantly different between the two groups (0.30 ± 0.06 versus 0.33 ± 0.09 mm, *p* = 0.195 and 1559 ± 217 versus 1612 ± 363 cm/s, *p* = 0.577, respectively) (Figure 1C,D).

### 3.3. Relationships of NT-proBNP with FMD, NID, bIMT and baPWV

**Figure 2:**
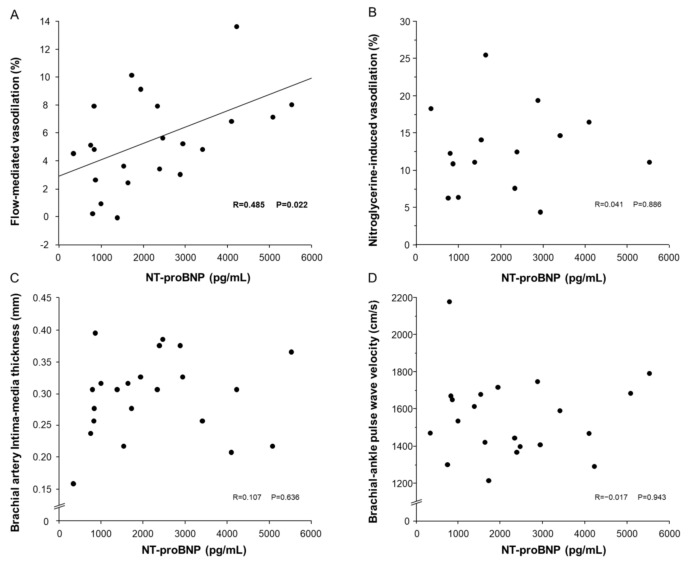
Association between N-terminal pro-brain natriuretic peptide (NT-proBNP) and flow mediated vasodilation (FMD). Scatter plots show the relationship of NT-proBNP with FMD (**A**) and nitroglycerine-induced vasodilation (**B**), brachial intima-media thickness (**C**) and brachial-ankle pulse wave velocity (**D**) in patients with wild-type transthyretin amyloid cardiomyopathy.

There was a significant relationship between NT-proBNP and FMD in patients with ATTRwt-CM (r = 0.485, *p* = 0.022) (Figure 2). There was no significant relationship of NT-proBNP with NID, bIMT or baPWV in patients with ATTRwt-CM (r = 0.041, *p* = 0.886; r = 0.107, *p* = 0.636 and r = −0.017, *p* = 0.943, respectively).

## 4. Discussion

In the present study, we demonstrated that FMD was significantly greater in patients with ATTRwt-CM than in control patients who were adjusted by vascular function confounding factors. Furthermore, we showed that there was a significant relationship between NT-proBNP and FMD in patients with ATTRwt-CM. To our knowledge, this is the first report showing endothelial function in patients with ATTRwt-CM.

Previous studies have shown that endothelial function is impaired in patients with HF, and endothelial dysfunction is also considered to be an independent risk factor of cardiovascular events in patients with HF [11,12]. In the present study, endothelial function was preserved and was augmented in patients with ATTRwt-CM compared with that in the matched controls. Plasma NT-proBNP levels were positively correlated with FMD. Our findings were contrary to the results of previous studies showing that endothelial function is impaired in patients with HF with reduced EF, patients with HF with mildly reduced EF and patients with HF with preserved EF [10,11,12,21]. We consider that possible reasons for the opposing results are related to the association of BNP with endothelial function. Natriuretic peptides (NPs) stimulate particulate guanylate cyclase (pGC) through the binding of NP receptor-A. Stimulation of pGC produces cyclic guanosine monophosphate (cGMP), leading to the regulation of physiological activity including inhibition of hypertrophy, fibrosis and apoptosis and anti-inflammation through activation of protein kinase G in the heart and vessels [22,23]. The NPs/pGC/cGMP pathway has protective effects on the cardiovascular system. Experimental studies have shown that BNP plays an important role in vascular function and vascular structure by suppressing inflammation and abnormal remodeling [23]. In a clinical study, intravenous infusion of nesiritide, a recombinant form of human BNP, improved FMD immediately after infusion and 24 h later in patients who underwent percutaneous coronary intervention [24]. TTR takes a long time to be deposited [25]. The protective effects BNP on the heart and vessels may contribute to the maintenance or augmentation of endothelial function in patients with ATTRwt-CM for a long time until the HF becomes severe.

Interestingly, BNP levels in patients with cardiac amyloidosis are different from those in typical HF patients. Takemura et al. [26] showed that BNP levels were higher in patients with cardiac amyloidosis than in control patients and they showed by immunohistochemistry of the myocardium that BNP expression in patients with cardiac amyloidosis was greater in the endocardial side than in the epicardial side and in myocytes neighboring deposited amyloid. This is because ventricular wall stress is stronger in the endocardial region than in the epicardial region, and amyloid deposition restricts myocyte movement and interferes with cooperative movement, resulting in regional mechanical stress. They concluded that both hemodynamic stress and regional mechanical stress increase BNP expression. In the present study, we also showed that there was an association of ventricular wall thickness with NT-proBNP in patients with ATTRwt-CM.

We consider that patients with ATTRwt-CM have a compensatory function to defend themselves by elevating BNP for a long period. None of the patients in this study died during the 596 ± 102 days of follow-up. However, two of the three patients with NYHA Ⅳ who were excluded from this study died during the study period. The FMD values of the two patients who died were −0.7% and 1.8% and the FMD of the other patient was 3.4%, suggesting that all three patients had low FMD. It is likely that, under the condition of advanced HF with NYHA IV, an increase in BNP cannot compensate for endothelial dysfunction associated with HF in patients with ATTRwt-CM.

The most common clinical symptom leading to a diagnosis of ATTR-CM is HF [9], but if the disease can be diagnosed early, before the onset of symptoms or at least while compensatory functions are still working, the prognosis may be improved by early initiation of TTR-specific therapies such as suppression of TTR synthesis, TTR stabilization, and TTR disruption/resorption [1,27,28,29]. Indeed, treatment with tafamidis, a TTR stabilizer, has been shown to improve prognosis compared to that in a placebo group and its effect is stronger in patients with NYHA I and II than in those with NYHA Ⅲ [30].

The establishment of ATTR-CM can take a long time to develop mechanisms of local compensatory effects because the deposition of amyloid fibrils derived from TTR is a gradual process [25]. Judging from the severity of hemodynamics, imaging, and functional capacity, ATTR-CM is stable for a relatively long period of time [1]. We consider that elevation of BNP is one of the specific compensatory mechanisms in patients with amyloidosis. Indeed, a large percentage of ATTR-CM patients without cardiac symptoms have high levels of biomarkers, such as troponin T and NT-proBNP, which are above the normal limits [31].

ATTRwt-CM is a disease with characteristics that differ from those of typical HF in terms of prognosis and medical therapy. In the present study, we were able to show that ATTRwt-CM is, likewise, different from typical HF judging from the aspect of endothelial function. ATTRwt-CM is an underestimated disease, and proper diagnosis is important. Diagnosis at an early stage is desirable.

ATTRwt-CM is a disease that typically affects elderly people, and patients with ATTRwt-CM included in our study were older than 70 years of age. We have clearly shown that FMD is lower in older age [32]. In this study, endothelial function assessment was performed under the conditions of ATTRwt-CM and aging, simultaneously. Therefore, patients with ATTRwt-CM had preserved endothelial function in comparison to age-matched patients without ATTRwt-CM, but still the value of FMD indicated impaired endothelial function because of aging.

There are some limitations in this study. First, this was a single-center study using a cross-sectional study design. Second, we included consecutive patients who were diagnosed with ATTRwt-CM and who agreed to have their FMD values measured. The number of subjects was relatively small. However, we have clearly shown that endothelial function is preserved in patients with ATTRwt-CM who do not have advanced HF. Third, as it was demonstrated by S. Yang et al. [33], race could have an impact on the association between NT-proBNP and FMD. The results for another race might be different. Finally, measurements of biochemical parameters of endothelial function in blood and urine samples would enable more specific conclusions concerning endothelial function in patients with ATTRwt-CM to be drawn.

## 5. Conclusions

Although it is true that the prognosis is very poor in patients with ATTR-CM after diagnosis and that patients with ATTRwt-CM who have an advanced stage of HF have endothelial dysfunction, endothelial function is preserved in patients with ATTRwt-CM who do not have advanced HF. Patients with ATTRwt-CM may have compensatory effects with respect to endothelial function through the elevation of BNP levels. We emphasize the clinical significance of early diagnosis and early treatment of ATTR-CM.

## Figures and Tables

**Table 1:** Clinical Characteristics of One-by-one Matched Controls and Patients with ATTRwt-CM.

Variables	Matched Controls (n = 22)	ATTRwt-CM (n = 22)	*p* Value
Age, yrs.	74 ± 11	77 ± 6	0.365
Men, n (%)	16 (72.7)	17 (77.3)	0.728
Body mass index, kg/m^2^	22.3 ± 3.2	22.4 ± 3.0	0.905
Heart rate, bpm	71 ± 15	71 ± 13	0.942
Systolic blood pressure, mmHg	115 ± 19	117 ± 19	0.678
Diastolic blood pressure, mmHg	70 ± 8	72 ± 7	0.505
Total cholesterol, mg/dL	179 ± 30	183 ± 38	0.682
Triglycerides, mg/dL	105 ± 51	113 ± 80	0.678
HDL-C, mg/dL	68 ± 20	71 ± 15	0.553
LDL-C, mg/dL	90 ± 25	89 ± 28	0.849
eGFR, mL/min/1.73 m^2^	66.4 ± 16.0	56.3 ± 19.6	0.072
Fasting blood glucose, mg/dL	120 ± 47	117 ± 34	0.860
Hemoglobin A1c, %	6.0 ± 0.7	6.0 ± 0.8	0.945
NT-pro BNP, pg/mL	470 ± 677	2202 ± 1478	<0.001
Medical history, n (%)			
Hypertension	7 (31.8)	7 (31.8)	1.000
Dyslipidemia	9 (40.9)	8 (36.3)	0.757
Diabetes mellitus	7 (31.8)	7 (31.8)	1.000
Hyperuricemia	6 (27.3)	7 (33.3)	0.665
Cerebrovascular disease	1 (4.8)	3 (14.3)	0.283
Current Smokers, n (%)	14 (66.7)	12 (54.6)	0.416
Medication, n (%)			
Calcium channel blockers	2 (9.5)	3 (13.6)	0.673
β blockers	7 (33.3)	9 (40.9)	0.607
ARBs/ACEIs	9 (42.9)	6 (27.3)	0.283
Statins	8 (36.4)	7 (31.8)	0.666

ATTRwt-CM indicates wild-type transthyretin amyloid cardiomyopathy; HDL-C, high-density lipoprotein cholesterol; LDL-C, low-density lipoprotein cholesterol; eGFR, estimated glomerular filtration rate; NT-pro BNP, N-terminal pro-brain natriuretic peptide; ARBs, angiotensin receptor blockers; ACEIs, angiotensin-converting enzyme inhibitors.

## Data Availability

The data presented in this study are available on request from the corresponding author. The data are not publicly available due to institutional policies requiring a data-sharing agreement.

## Supplementary Materials

The following supporting information can be downloaded at: https://www.mdpi.com/article/10.3390/jcm12072534/s1, Table S1: Clinical Characteristics of all Subjects; Table S2. Echocardiographic Parameters of One-by-one Matched Controls and Patients with ATTRwt-CM.
