# Supplementary material for: Endothelial Function Is Preserved in Patients with Wild-Type Transthyretin Amyloid Cardiomyopathy
Source: J Clin Med. 2023 Mar 27;12(7):2534. doi: 10.3390/jcm12072534 (PMC10095569; doi:10.3390/jcm12072534)
Supplement: Supplementary file 1 [file jcm-12-02534-s001.zip › jcm-2266397-supplementary.pdf]

Online Supplemental Data

Endothelial Function Is Preserved in Patients with Wild-type Transthyretin Amyloid Cardiomyopathy

Yu Hashimoto [1], Takayuki Yamaji [1], Toshiro Kitagawa [1], Yukiko Nakano [2], Masato Kajikawa [2], Kenichi Yoshimura [3], Kazuaki Chayama [4], Chikara Goto [5], Syunsuke Tanigawa [6], Aya Mizobuchi [6], Takahiro Harada [6], Farina Mohamad Yusoff [6], Shinji Kishimoto [6], Tatsuya Maruhashi [6], Asuka Fujita [6,7], Toshio Uchiki [6,7], Ayumu Nakashima [8] and Yukihito Higashi [2,6,*]

[1] Department of Cardiovascular Medicine, Graduate School of Biomedical Sciences, Hiroshima University, Hiroshima 734-8551, Japan
[2] Division of Regeneration and Medicine, Medical Center for Translational and Clinical Research, Hiroshima University Hospital, Hiroshima 734-8551, Japan
[3] Department of Biostatistics, Medical Center for Translational and Clinical Research, Hiroshima University Hospital, Hiroshima 734-8551, Japan
[4] Collaborative Research Laboratory of Medical Innovation, Hiroshima University, Hiroshima 734-8551, Japan
[5] Department of Physical Therapy, Hiroshima International University, Hiroshima 737-0112, Japan
[6] Department of Regenerative Medicine, Research Institute for Radiation Biology and Medicine, Hiroshima University, Hiroshima 739-8529, Japan
[7] Plastic and Reconstructive Surgery, Hiroshima University Hospital, Hiroshima 734-8551, Japan
[8] Department of Stem Cell Biology and Medicine, Graduate School of Biomedical and Health Sciences, Hiroshima University, Hiroshima 734-8551, Japan

*** Address for correspondence:** Yukihito Higashi, MD, PhD, FAHA
Department of Regenerative Medicine,
Research Institute for Radiation Biology and Medicine, Hiroshima University
1-2-3 Kasumi, Minami-ku, Hiroshima 734-8551, Japan
Phone: +81-82-257-5831 Fax: +81-82-257-5831
E-mail: yhigashi@hiroshima-u.ac.jp

Supplemental Methods

Measurement of flow mediated vasodilation and nitroglycerine-induced vasodilation

A high-resolution linear artery transducer was coupled to computer-assisted analysis software (UNEXEF18G, UNEX Co., Nagoya, Japan) that used an automated edge detection system for measurement of the brachial artery diameter. A blood pressure cuff was placed around the forearm of each subject. The brachial artery was scanned longitudinally 5 to 10 cm above the elbow. When the clearest B-mode image of the anterior and posterior intimal interfaces between the lumen and vessel wall was obtained, the transducer was held at the same point throughout the scan by using a special probe holder (UNEX Co.) to ensure consistency of the imaging. Depth and gain settings were set to optimize the images of the arterial lumen wall interface. When the tracking gate was placed on the intima, the artery diameter was automatically tracked, and the waveform of diameter changes over the cardiac cycle was displayed in real time using the flow mediated vasodilation (FMD) mode of the tracking system. This allowed the ultrasound images to be optimized at the start of the scan and the transducer position to be adjusted immediately for optimal tracking performance throughout the scan. Pulsed Doppler flow was assessed at baseline and during peak hyperemic flow, which was confirmed to occur within 15 seconds after cuff deflation. Blood flow velocity was calculated from the color Doppler data and was displayed as a waveform in real time. Baseline longitudinal images of the artery were acquired for 30 seconds, and then the blood pressure cuff was inflated to 50 mm Hg above systolic pressure for 5 minutes. The longitudinal image of the artery was recorded continuously until 5 min after cuff deflation. Pulsed Doppler velocity signals were obtained for 20 sec at baseline and for 10 sec immediately after cuff deflation. Changes in brachial artery diameter were immediately expressed as percentage change relative to the vessel diameter before cuff inflation. FMD was automatically calculated as the percentage change in peak vessel diameter from the baseline value. Percentage of FMD [(Peak diameter - Baseline

diameter)/Baseline diameter] was used for analysis. Blood flow volume was calculated by multiplying the Doppler flow velocity (corrected for the angle) by heart rate and vessel cross-sectional area (-r^2). Reactive hyperemia was calculated as the maximum percentage increase in flow after cuff deflation compared with baseline flow.

The response to nitroglycerine was used for assessment of endothelium-independent vasodilation (NID). After acquiring baseline rest images for 30 seconds, a sublingual tablet (nitroglycerine, 75 µg) was given, and imaging of the artery was done continuously for 5 minutes. NID was automatically calculated as a percentage change in peak vessel diameter from the baseline. Percentage of NID [(Peak diameter - Baseline diameter)/Baseline diameter] was used for analysis. Inter- and intra-coefficients of variation for the brachial artery diameter were 1.6% and 1.4%, respectively, in our laboratory.

Measurement of brachial artery intima-media thickness

Measurement of brachial artery intima-media thickness (bIMT) was automatically performed on A-mode images of the far wall of the brachial artery. The analysis system automatically chose the measurement point where an image of the posterior intimal interface was clearly obtained. If the measurement point was inappropriate, another clear image site could be manually selected for measurement. A total of 21 points over a 3-mm length of IMT in the 10-mm longitudinal image depicted in the analysis display were measured and the mean value per image was automatically calculated. bIMT was measured at the same point in each image. The average of mean values obtained from 10 cardiac cycles was defined as bIMT. When measuring carotid IMT, we used an anatomical landmark such as the carotid-artery bulb. Measurement of bIMT was performed at the proper site where the clearest B-mode image of the anterior and posterior intimal interfaces between the lumen and vessel wall was obtained at 5 to 10 cm above

the elbow. The coefficients of variation of intra- and inter-observer bIMT measurements were 3.1% and 4.0%, respectively.

Measurement of brachial-ankle pulse wave velocity

Briefly, brachial-ankle pulse wave velocity (baPWV), an index of arterial stiffness, was determined by using two pressure sensors placed on the right ankle and left brachial arteries to record each pulse wave simultaneously, and the time lag (*t*) between the notches of the two waves, using a pulse wave velocimeter (Form PWV/ABI, model BP-203RPE, Colin Co.). The distance (*D*) between the two recording sensors was calculated automatically by inputting the value of individual height. The PWV value was calculated as PWV=*D*/t. PWV was measured for five consecutive pulses, and averages were used for analysis.

Echocardiography

Echocardiograms were obtained by using commercially available equipment (Vivid E95, GE). Routine two-dimensional imaging examinations were performed in parasternal long and short axis views and apical two and four chamber views. Echocardiographic parameters were evaluated by mitral inflow velocities, pulmonary venous flow velocities, mitral annulus velocities from Doppler tissue imaging, and left atrial (LA) volume. After the mitral inflow velocity had been traced, early (E) and late (A) diastolic mitral inflow velocities, deceleration time of E velocity, and the ratio of early to late peak velocities (E/A) were obtained. In addition, pulmonary venous systolic and diastolic flow velocities were obtained. For measurement of mitral annulus flow velocity, sample volume was located at the septal side of the mitral annulus, and early (E’) and late (A’) diastolic mitral annulus velocities were measured. The ratio of early diastolic mitral inflow velocity to late diastolic mitral annulus velocity (E/E’) was obtained. LA

volume was measured by the biplane area-length formula and indexed for body surface area. Left ventricular (LV) mass was calculated according to the Penn Convention. LV end-diastolic volume index was calculated by the modified Simpson formula.

Table S1. Clinical Characteristics of all Subjects

Variables	Total (n=3814)	Without ATTRwt-CM (n=3792)	With ATTRwt-CM (n=22)	P value
Age, yr	57 ± 19	56 ± 19	77 ± 6	<0.001
Men, n (%)	2290 (60.0)	2278 (60.1)	17 (77.3)	0.100
Body mass index, kg/m^2	23.6 ± 4.0	23.6 ± 4.0	22.4 ± 3.0	0.170
Heart rate, bpm	70 ± 12	70 ± 12	71 ± 13	0.674
Systolic blood pressure, mmHg	128 ± 19	128 ± 19	117 ± 19	0.005
Diastolic blood pressure, mmHg	76 ± 13	76 ± 13	72 ± 7	0.136
Total cholesterol, mg/dL	192 ± 38	192 ± 38	183 ± 38	0.327
Triglycerides, mg/dL	141 ± 104	142 ± 104	113 ± 80	0.242
HDL-C, mg/dL	59 ± 17	59 ± 17	71 ± 15	0.004
LDL-C, mg/dL	106 ± 34	106 ± 34	89 ± 28	0.032
eGFR	61.2 ± 18.4	66.4 ± 16.0	56.3 ± 19.6	0.072
Uric acid, mg/dL	5.7 ± 1.5	5.7 ± 1.5	6.7 ± 2.2	0.002
Fasting blood glucose, mg/dL	113 ± 37	113 ± 37	117 ± 34	0.663
Hemoglobin A1c, %	6.0 ± 0.9	6.0 ± 0.9	6.0 ± 0.8	0.734
NT-pro BNP, pg/mL	470 ± 1422	450 ± 1455	2202 ± 1478	<0.001
Medical history, n (%)				
Hypertension	2519 (66.7)	2512 (66.9)	7 (31.8)	<0.001
Dyslipidemia	2052 (54.3)	2044 (54.5)	8 (36.4)	0.089
Diabetes mellitus	918 (24.3)	911 (24.3)	7 (31.8)	0.410
Hyperuricemia	729 (19.6)	722 (19.5)	7 (33.3)	0.110
Cardiovascular disease, n (%)	284 (7.6)	281 (7.5)	3 (14.3)	0.244
Current Smokers, n (%)	1776 (47.4)	1764 (47.3)	12 (54.6)	0.500
Medication, n (%)				
Calcium channel blockers	1677 (47.2)	1674 (47.4)	3 (13.6)	<0.001
β blockers	458 (12.9)	449 (12.7)	9 (40.9)	<0.001
ARB/ACEIs	1336 (37.6)	1330 (37.6)	6 (27.3)	0.317
Statins	1164 (32.7)	1157 (32.7)	7 (31.8)	0.927

ATTRwt-CM indicates wild-type transthyretin amyloid cardiomyopathy; HDL-C, high-density lipoprotein cholesterol; LDL-C, low-density lipoprotein cholesterol; eGFR, estimated glomerular filtration rate; NT-pro BNP, N-terminal pro-brain natriuretic peptide; ARB, angiotensin receptor blockers; ACEIs, angiotensin-converting enzyme inhibitors.

Table S2. Echocardiographic Parameters of One-by-one Matched Controls and Patients with ATTRwt-CM

Echocardiographic parameters	Matched controls (n=22)	ATTRwt-CM (n=22)	P value
LV ejection fraction, %	60.7 ± 12.9	50.7 ± 15.2	0.053
LV ejection fraction < 50, %	9.1	45.5	0.037
LA diameter, mm	39.0 ± 6.1	42.0 ± 6.1	0.196
LA volume index, ml/m^2	35.2 ± 5.2	49.4 ± 12.6	0.009
Interventricular septal thickness, mm	9.5 ± 2.0	14.7 ± 3.6	<0.001
Posterior LV wall thickness, mm	10.0 ± 1.8	13.9 ± 2.1	<0.001
LV end-diastolic diameter, mm	46.4 ± 7.9	42.7 ± 4.3	0.083
LV end-systolic diameter, mm	31.7 ± 9.2	32.1 ± 4.9	0.853
LV end-diastolic diameter index, mm	30.6 ± 4.8	26.7 ± 3.3	0.013
LV end-systolic diameter index, mm	20.7 ± 5.1	20.2 ± 3.9	0.756
LV end-diastolic volume, ml	87.0 ± 43.9	76.5 ± 21.1	0.373
E velocity, cm/s	58.5 ± 11.5	73.8 ± 21.7	<0.001
A velocity, cm/s	67.3 ± 28.8	42.4 ± 21.6	<0.001
E/A	1.0 ± 0.6	2.0 ± 1.4	<0.001
Deceleration time, ms	202 ± 36.5	195 ± 43	0.643
Septal A’ velocity, cm/s	7.6 ± 1.5	4.3 ± 1.5	<0.001
Septal E’ velocity, cm/s	5.2 ± 1.7	3.6 ± 1.0	<0.001
Lateral A’ velocity, cm/s	9.3 ± 2.9	5.1 ± 2.5	<0.001
Lateral E’ velocity, cm/s	6.8 ± 2.3	5.1 ± 1.7	<0.001
Septal E/E’ ratio	11.9 ± 3.4	22.1 ± 9.4	<0.001
Lateral E/E’ ratio	9.0 ± 3.2	16.5 ± 10.1	<0.001

ATTRwt-CM indicates wild-type transthyretin amyloid cardiomyopathy; LV, left ventricular, LA, left atrial; E, early diastolic mitral inflow; A, late diastolic mitral inflow; A’, late diastolic mitral annulus velocity; E’, early diastolic mitral annulus velocity.
